# Brainard Medical Society

**Published:** 1873-10

**Authors:** J. W. C. Eaton

**Affiliations:** Sec’y.


### Reports of Societies.

### *Brainard Medical Society.*

The Brainard Medical Society met in the Christian Chapel, in Winamac, Ind., June 12, 1873.

Members present: F. B. Thomas, James Thomas, A. R. Thompson, W. T. Cleland, G. W. Nafe, Le Roy Rogers, Wm. Kelsey, D. F. Moss, H. E. Pattison, W. H. Thompson, G. W. Thompson, I. B. Washburn, and J. W. C. Eaton.

The President, Dr. Glazebrook, being absent, the Secretary called the Society to order, and introduced the President elect, Dr. I. B. Washburn, of Logansport.

Before taking the Chair, he read his inaugural address, which elicited the closest attention. A copy of it was requested for publication in the Winamac and Logansport papers.

On motion of Dr. Kelsey, T. J. Loring, medical student, was elected a member of the Society.

Dr. Eaton, of Pulaski, read a paper on the Pathology of Typhoid Fever.

In his paper, Dr. Eaton objected to the term typhoid, but said the typhoid state would be preferable. He said the products of tissue metamorphosis are chiefly eliminated by the kidneys. As long as the kidneys are equal to the increased work, the blood is properly depurated and the typhoid state warded off. But if, from the large amount of effete matter, or from any other cause, the kidneys be not equal to the task, the blood becomes poisoned, and either convulsions or the typhoid state supervene.

The paper was discussed at length by Drs. Cleland, Kelsey, Rogers, Pattison, Nafe, and Washburn.

The Society adjourned to meet in Star City, August 7, 1873.

J. W. C. Eaton, *Sec'y.*

